# Route of inoculation influences *Trypanosoma congolense* and *Trypanosoma brucei brucei* virulence in Swiss white mice

**DOI:** 10.1371/journal.pone.0218441

**Published:** 2019-06-20

**Authors:** Kariuki Ndungu, Daniel Thungu, Florence Wamwiri, Paul Mireji, Geoffrey Ngae, Purity Gitonga, James Mulinge, Joanna Auma, John Thuita

**Affiliations:** 1 Biotechnology Research Institute, Kenya Agricultural and Livestock Research Organization, Kikuyu, Kenya; 2 Centre for Geographic Medicine Research—Coast, Kenya Medical Research Institute, Kilifi, Kenya; 3 Food Crops Research Institute, Kenya Agricultural and Livestock Research Organization, Nairobi, Kenya; 4 Kenya Methodist University, Nairobi, Kenya; 5 Meru University of Science and Technology, Meru, Kenya; Academic Medical Centre, NETHERLANDS

## Abstract

Experiments on infections caused by trypanosomes are widely performed in Swiss white mice through various inoculation routes. To better understand the effect of route of trypanosome inoculation on disease outcomes in this model, we characterised the virulence of two isolates, *Trypanosoma bruce*i KETRI 2710 and *T*. *congolense* KETRI 2765 in Swiss white mice. For each of the isolates, five routes of parasite inoculation, namely intraperitoneal (IP), subcutaneous (SC), intramuscular (IM) intradermal (ID) and intravenous (IV) were compared using groups (n = 6) of mice, with each mouse receiving 1x10^4^ trypanosomes. We subsequently assessed impact of the routes on disease indices that included pre-patent period (PP), parasitaemia levels, Packed Cell Volume (PCV), bodyweight changes and survival time. Pre-patent period for IP inoculated mice was a mean ± SE of 3.8 ± 0.2 and 6.5 ± 0.0 for the *T brucei* and *T*. *congolense* isolates respectively; the PP for mice groups inoculated using other routes were not significantly different(p> 0.05) irrespective of route of inoculation and species of trypanosomes. With ID and IP routes, parasitaemia was significantly higher in *T*. *brucei* and significantly lower in *T*. *congolense* infected mice and the progression to peak parasitaemia routes showed no significant different between the routes of either species of trypanosome. The IM and ID routes in *T*. *congolense* inoculations, and IP and IV in *T*. *b*. *brucei* induced the fastest and slowest parasitaemia progressions respectively. There were significant differences in rates of reduction of PCV with time post infection in mice infected by the two species and which was more pronounced in sc and ip injected mice. No significant differences in mice body weight changes and survivorship was observed between the routes of inoculation. Inoculation route therefore appears to be a critical determinant of pathogenicity of *Trypanosoma congolense* and *Trypanosoma brucei brucei* in murine mouse model of African trypanosomiasis.

## Introduction

African trypanosomes are extracellular protozoan parasites that cause chronic infections in Humans and their livestock, and are predominantly transmitted by tsetse fly[1]. *Trypanosoma congolense and Trypanosoma brucei brucei*, are among the main trypanosomes, which causes livestock infections [2,3].Available data on *T*. *congolense* and *T*. *b*. *brucei* pathogenicity suggest *T*. *congolense* as the most pathogenic trypanosome in cattle [4] and has been shown to exhibit greater virulence in experimental mice [5]. In contrast, *T*. *b*. *brucei* is particularly virulent in dogs, camels and horses, with the horse often succumbing to infection within a few months in the absence of treatment[6] The pathogenicity of these parasites has typically been determined through intraperitoneal (IP) inoculation[7,8] of Swiss white mice animal models of trypanosomiasis[9] Parasitaemia, live body weight, packed cell volume (PCV) and survivorship in the mice have been considered appropriate indicators of the pathogenicity[5] [10].The various routes of inoculation including intraperitoneal (IP),subcutaneous(SC), intramuscular (IM), intravenous (IV) and intradermal (ID) have, previously been used to infect mice with trypanosomes[11,12,13] The IP inoculation is normally administered through the abdominal wall into the peritoneal cavity of the mice. Consequently, there is no visual confirmation of inoculation as opposed to other comparable methods such as intravenous (IV) or intradermal (ID) routes [14] While IV has been used as an inoculation route, the technique exposes mice to higher risks of infection by other pathogens [15] and tissue irritation in mice. Similarly, intramuscular (IM) route has an advantage of rapid and uniform deposition of the inoculum[15] but institutes muscle necrosis or inflammation of the nerves that can lead to lameness and self-mutilation of the affected area[16,17].Administration of ID inoculation is complicated by thin skin in mice that necessitates anesthetization and the fur clipping of the mice[18] with inadvertent SC administration as a common complication [7].In this study, we the compared the impact of route of inoculation of *T*. *congolense* KETRI 2765 or *T*. *b*. *brucei* KETRI 2710 parasites in Swiss white mice on pathogenicity of the parasites.

### Ethics

All protocols and procedures used in this study were reviewed and approved by the Biotechnology Research Institute Kenya Agricultural and Livestock Research Organization (BioRI-KALRO-) Institutional Animal Care and Use Committee (IACUC).The IACUC approval number is C/BioRI/4/325/II/47

## Materials and methods

### Experimental animals and parasites

Swiss white male mice were used in this study. The mice (25-30g) were obtained from BioRI-KALRO Small Animals Breeding Unit, maintained on mice pellets (Unga Feeds Ltd, Kenya) and clean water *ad libitum*, and acclimatized to experimental room conditions for two weeks. During this period, the mice were dewormed by intraperitonial injection using Ivermectin (Noromectin) at 0.2mg/kg as described by[19] to potentially eliminate possible ecto- and endoparasites in the mice. *Trypanosoma congolense* KETRI 2765[20] (heterogeneous population) and *Trypanosoma brucei brucei* KETRI 2710 [21] (clonal population) trypanosome strains were used in this study. The *T*. *congolense* KETRI 2765 was originally isolated from bovine in 1980 in Baringo Kenya while *T*. *b*. *brucei* KETRI 2710 was donated to the KALRO-BioRI cryobank by International Livestock Research Institute ILRI (ILRAD 603).

### Swiss white mice trypanosome infections and pathogenicity experiments

The parasites (*T*. *brucei* or *T*. *congolense*) were separately expanded in two donor Swiss white mice immuno-suppressed with cyclophosphamide at 100mg/kg/day for three consecutive days (300mg/kg total dose) [22] The donor mice were examined daily for parasitaemia. At peak parasitaemia (1.3x10^8^ trypanosomes/ml for the *T*. *congolense* and 5.0x10^8^trypanosomes/ml for the *T*. *b*. *brucei)*, the donor mice were anaesthetized using carbon dioxide, the parasites collected through cardiac puncture in tubes containing 10% EDTA and quantified using improved Neubauer chamber method of [23] The parasites (in blood) were subsequently diluted to 1.0 x 10^4^ trypanosomes/mL with Phosphate-buffered-Saline-Glucose (PSG) (44 mM NaCl, 57 mM Na_2_HPO_4_, 3 mM KH_2_PO_4_, 55 mM glucose) pH 8.0 solution. Subsequently, five groups of six mice [5,24,25]each were intraperitoneally, subcutaneously, intramuscularly, intradermaly or intravenously inoculated with 0.1mL (1.0 x 10^4^ trypanosomes) of the *T*.*brucei* or *T*. *congolense* infected blood in PSG, constituting 60 (6 x 2x 5) individual experiments. Base line data on packed cell volume (PCV) and body weight changes were collected for a period of 14 days before inoculation. Six mice were similarly handled but were not inoculated with any of the parasites and therefore constituted the control group for our inoculated groups. Parasitaemia in the inoculated groups were scored daily in each individual mouse using the rapid matching method of [26] from blood collected from mice using the tail amputation method[27]. Packed cell volume (PCV) was determined weekly using microhaematocrit method [5] from blood collected from the tail vein into heparinized capillary tubes as described by[28] Each individual mouse was weighed weekly. Survival of individual mice was monitored daily where a mouse was considered *at extremis* and withdrawn from the study if the PCV declined by 25% or more and/or has high terminal parasitaemia (1x10^9^/mL) for at least two consecutive days[10] Such mice were euthanized by CO2 asphyxiation in accordance with guidelines of the Institutional Animal Care and Use Committee (IUCAC) and as described by [29]. Mice surviving beyond 45 days post infection were similarly euthanized and their survival time categorized as censored data. At termination of the experiment, all mice carcasses were wrapped with polythene paper, kept in a freezer and then disposed as per BioRI-KALRO Institutional IUCAC guidelines. Overall all inoculated mice were monitored for 45 days post inoculation, which constituted the experimentation duration.

### Data analysis

Differences in pre-patent periods and effect of inoculation routes and trypanosome species on peak parastemia were evaluated using two way analysis of variance (ANOVA) with inoculation routes and parasite species as factors. Means that were significantly different were identified using Tukey’s HSD post hoc analysis[30]. Differences in rates of increase in *T*. *congolense* or *T*.*b brucei* parasitaemia following inoculations through various routes were established by comparing effective median times (ET50) for each inoculation route using Probit analysis. Effects of different inoculation routes on PCV and bodyweights were analysed through linear regression analysis of the changes in weight of individual mice against time (days). Impact of the different of inoculation routes on survivorship of *T*. *congolense* or *T*. *b brucei* were analyzed by Kaplan-Meier method to determine survival distribution [31]function with log-rank (Mantel-Cox) and Gehan-Breslow-Wilcoxon test[32] to provide insight on effect of route of trypanosome infection on survivorship. All analyses were conducted using GraphPad Prism version 7.00 for Mac (GraphPad Software, La Jolla California USA).

## Results

**Fig 1:**
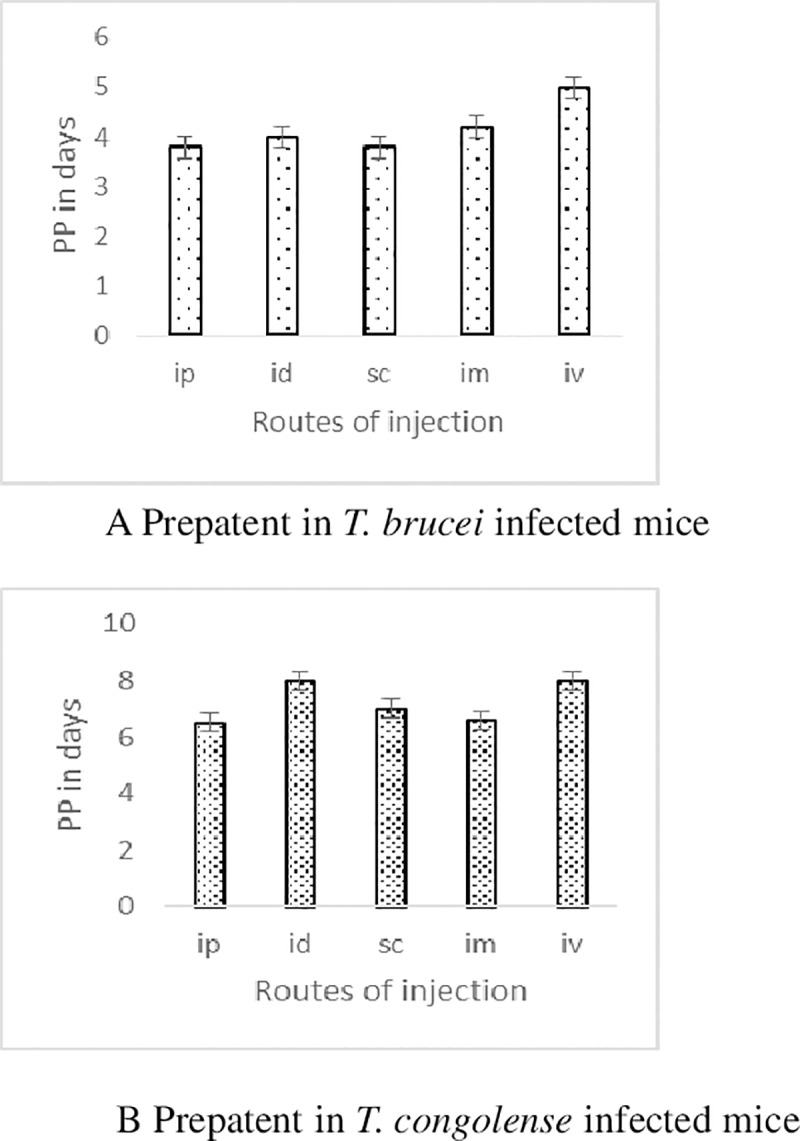
Mean Prepatent period (10–90%) of *T*. *congolense* or *T*. *b*. *brucei* infection in Swiss white mice following infections through various routes.

**Fig 2:**
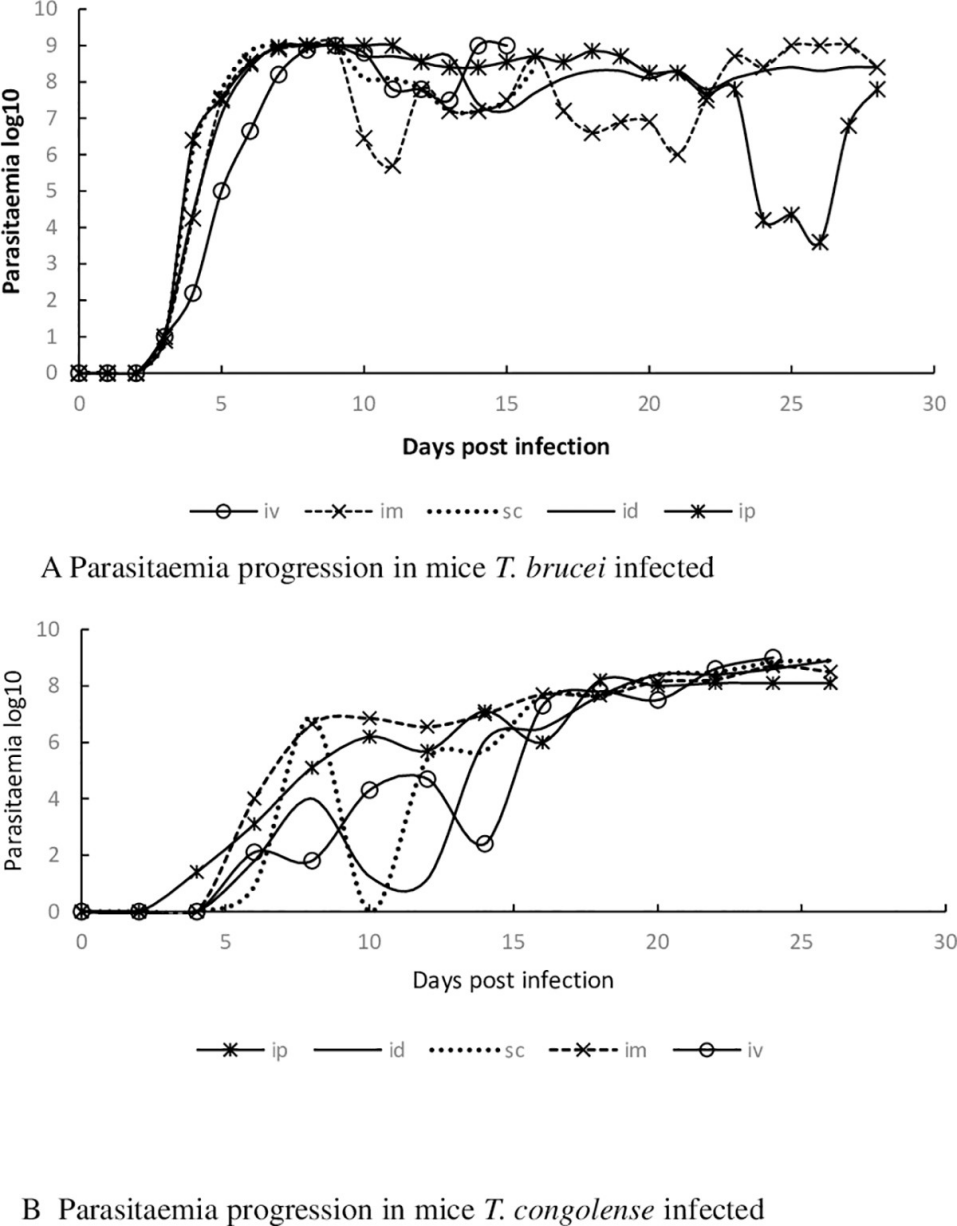
Parasitaemia progression in mice infected with *T*. *brucei* and *T*. *congolense*.

**Fig 3:**
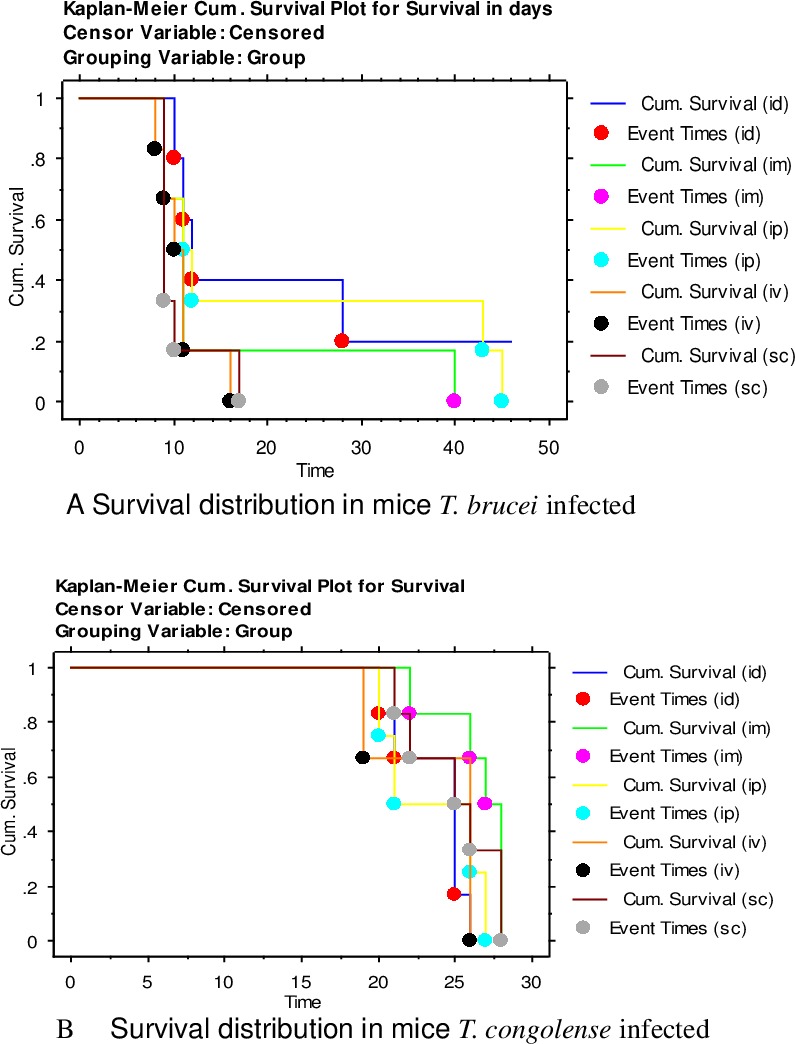
Kaplan -Meir cumulative survival plots for mice inoculated with *T*. *congolense* or *T*. *b*.*brucei* through various routes and their respective controls.

A total of 3/6(50%) and 4/6 (67%) of the mice inoculated with *T congolense* via IV and IP routes and all (n = 6) mice inoculated via IM, SC and ID developed patent parasitaemia n. Similarly, all (n = 6) mice inoculated with *T*. *b*. *brucei* via IP, IM, SC and IV. Most (5/6) of the mice inoculated via ID route developed parasitaemia. Overall mean pre-patent periods in *T*. *congolense* infections were between 5.8 and 8.0 days while the same in *T*. *b*. *brucei* ranged between 3.8 and 5.0 days (Fig 1 (i) & (ii)). The pre-patent periods within mice infected with either parasite were similar, irrespective of the routes on inoculation (F4, 44 = 2.03, P = 0.1067), our analysis revealed that the overall peak parasitaemia in either species was not significantly influenced by the route of inoculation (F4, 48 = 0.7201, P = 0.5824). The mode of inoculation significantly influenced the progression (ET50) of *T*. *congolense* (F4, 637 = 32.11, P = <0.0001) or *T*. *b*. *brucei (*F4, 382 = 8.903, P = <0.0001) infections in mice. The IM and ID routes in *T*. *congolense* inoculations, and IP and IV in *T*. *b*. *brucei* induced the fastest and slowest parasitaemia progressions respectively (Fig 2 (i) & (ii)). There were significant differences in rates of reduction of PCV (F5, 14 = 4.632, P = 0.0105) with time post infection in mice infected by the two species and which was more pronounced in sc and ip injected mice (Table 1) (F 1, 3 = 10.36, P = 0.0486). However, no significant differences in bodyweight was observed among different routes of injection (Table 2). The routes of inoculation significantly influenced survivorship in mice inoculated by *T*. *congolense* (Χ^2^ (df = 5) = 11.35, p = 00449) or *T*. *b*. *brucei* (Χ^2^ (df = 5) = 18.83, p = 0021), with *T*. *congolense* inoculated mice living longer (Fig 3 (i)&(ii)). However, there was no significant difference between the routes of either group.

**Table 1:** Mean temporal changes in Parked Cell Volumes (PCV) in Swiss white mice following various routes of inoculation with *T*. *b*. *brucei* or *T*. *congolense* parasites.

		Days Post Inoculation				
Trypanosome Species	Inoculation Route	0	7	14	21	28
No Inoculation Control	None	30.8 ± 0.8	30.3 ± 1.0	29.2 ± 0.9	29.2 ± 0.9	30.0 ± 0.9
*T*. *b*. *brucei*	Intraperitonial	28.3 ± 1.0	28.3 ± 0.9	29.5 ± 0.5	32.5 ± 0.5	31.0 ± 0.0
	Subcutaneous	27.3 ± 0.6	26.8 ± 0.4	21.0 ± 0.0	-	-
	Intramuscular	28.8 ± 1.0	28.3 ± 1.2	27.0 ± 0.0	28.0 ± 0.0	30.0 ± 0.0
	Intradermal	29.6 ± 0.4	29.2 ± 0.9	26.5 ± 1.5	30.5 ± 1.5	33.0 ± 0.0
	Intravenous	29.7 ± 0.4	28.7 ± 0.6	30.0 ± 0.0	-	-
*T*. *congolense*	Intraperitonial	26.8 ± 0.9	26.5 ±1.0	29 ± 1.2	30.5 ± 1.5	36.0 ± 0.0
	Subcutaneous	27.7 ± 0.8	27.7 ± 0.7	28.8 ± 0.8	29.2 ± 0.8	28.0 ± 0.0
	Intramuscular	28.7 ± 1.1	28.2 ± 0.9	30 ± 1.0	32.5 ± 1.3	29.7 ± 1.5
	Intradermal	28.5 ± 0.6	28.3 ± 0.7	29.2 ± 0.4	30.3 ± 0.6	32.0 ± 0.0
	Intravenous	29.7 ± 0.9	30 ± 1.0	31.3 ± 1.2	30.5 ± 0.5	-

Numbers in parenthesis represent number of mice alive. Where only one mouse is remaining, % change is not indicated.

**Table 2:** Mean body weight changes in Swiss white mice following various routes of inoculation with *T*. *b*. *brucei* or *T*. *congolense* parasites.

		Days Post Inoculation				
Trypanosome Species	Inoculation Route	0	7	14	21	28
No Inoculation Control	None	52.5 ± 1.2(6)	53.7 ±1.6 (6)	52.3 ±12.0 (6)	52.0 ± 0.4(6)	50.6 ± 1.1
*T*. *b*. *brucei*	Intraperitonial	51.7 ± 1.7 (6)	47.7 ± 2.3 (6)	46.5 ± 4.5(2)	45.5 ± 0.5 (2)	45.5 ± 1.5
	Subcutaneous	51.0 ± 0.4 (6)	51.0 ± 0.9 (6)	19.0 ± 0.0 (1)	-	-
	Intramuscular	52.8 ± 9(6)	51.7 ± 1.4 (6)	38.0 ± 0.0 (1)	48.0 ± 0.0 (1)	45.0 ± 0.0
	Intradermal	53.0 ± 0.4 (6)	47.5 ± 1.0 (6)	39.5 ± 2.5(3)	47.0 ± 3.0 (3)	50.0 ± 0.0
	Intravenous	50.5 ± 1.4(6)	49.3 ± 1.3 (6)	32 .0± 0.0 (1)	-	-
*T*. *congolense*	Intraperitonial	55.0 ± 0.6(4)	52.5 ± 1(4)	51.5 ± 0.6(4)	46.0 ± 0.0(2)	51.0 ± 0.0
	Subcutaneous	54.2 ±0.5(6)	50.2 ± 1.3(6)	50.3 ± 1.1(6)	43.0 ± 1.0 (5)	40.0 ± 0 .0
	Intramuscular	53.7 ± 0.7(6)	48.5 ± 1.5(6)	51.3 ± 0.6(6)	42.0 ± 1.5 (6)	36.3 ± 3.0
	Intradermal	54.0 ± 0.9(6)	50.2 ± 0.5(6)	52.5 ± 0.6(6)	43.0 ± 2.5(4)	47.0 ± 0.0
	Intravenous	55.0 ± 0.6(3)	50.7 ± 1.5(3)	48.3 ± 2.9(3)	48.0 ± 1.0 (3)	-

## Discussion

In this study, we investigated the effect of intraperitonial (IP), subcutaneous (SC), intramuscular (IM), intravenous (IV) and intradermal (ID) routes of inoculation on the pathogenesis of *T*. *congolense* or *T*. *brucei* trypanosomes infection in the murine model. Currently, the IP route is the most commonly used method of inoculation of trypanosomes into mice [14], although the other routes are available for use[11,12] [13]. In our current study, we obtained a complete 6/6 infection success rate for all groups with the exception of IP- *T congolense* group which had 4/6 (67%), IV -*T congolense* group with 3/6 (50%), and ID-*T*. *brucei* group with 5/6 (83%) demonstrating the effectiveness of these routes in experimental trypanosomiasis. However, the failure of some mice to develop patent parasitaemia through the IP, IV and ID routes was probably as a result of the limitations associated with these routes of injection. Previous authors have reported the IP injection route to have a failure rate of the order of 10–20% probably brought about by the penetration of the needle into urinary bladder, intestines, fat or muscles[33] The intravenous injection route in mice, unlike in larger mammals, is difficult to execute due to the small size of the blood vessels, resulting in misadministration of the inocula as reported by[34]. The failure observed in *T*. *brucei* ID infected mice may be attributed to resistance. Indeed, previous studies have shown Mice being 100 times more resistant to intradermal infections by *Trypanosoma congolense* or *Trypanosoma brucei* than to intraperitoneal infections [13]. Our results further showed that the pre-patent period ranged between 5.8 and 8.0in *T*. *congolense* infected mice, and between 3.8 and 5.0 days in *T*. *brucei* infected mice, and were a not significantly different suggesting that neither of the route is superior for studies on the incubation period of the two species of trypanosomes. The rate of substance absorption with SC route has been reported to be lower compared with other parenteral routes [35], the significance delay with *T*. *congolense* infections may attributed to the combined effect of both the NO and other interferons. Indeed, IFN- plays a role in resisting *T*. *b*. *rhodesiense* infection [36] whereas IFN- and NO together with the antibody response have been shown to be crucial in the control of *T*. *congolense i*nfection [37].We further observed that the mode of inoculation influenced the parasitaemia progression with IM and ID routes in *T*. *congolense* infections and IP and IV routes in *T*. *brucei infections* inducing fastest and slowest parasitaemia progression respectively. While the slow progression in ID *T*. *congolense* infected mice may be attributed to the resistance associated with this route as reported elsewhere[13], it is not clear why route IM influences the parasitaemia progression in *T*. *congolense* necessitating further investigation. Our finding is important as it provides the IM and IP as the ideal routes of inoculation in murine *T*. *congolense* and *T*. *b*. *brucei* experiments requiring high parasitaemia respectively. Data from our study showed occurrence of anaemia in mice infected with *T*. *congolense* or *T*. *brucei* irrespective of the route of inoculation and in agreement with previous observation[38]. However, the decline in PCV was not significant suggesting all the routes are ideal for inoculation in studies on anaemia induced by either of these species of trypanosomes. Interestingly, the decline was not significant in the IM inoculated mice and which demonstrated significantly faster rate of parasitaemia progression. Our data is important as it confirms previous observation showing parasitaemia does not translate to severe anaemia[39]. The ID route is however limited by the possibility of inadvertent subcutaneous administration a common complication of intradermal injections[7]. Data from our study showed that the route of inoculation influenced the survival of the infected mice. However, there was no significant difference between the routes of inoculation in mice infected by the two species of trypanosomes. Data from previous studies have associated survival to virulence [5] [40]. Our observation is therefore important as it shows that none of the routes is superior in virulence studies using the two species of trypanosomes. In conclusion, our study showed that all the routes are suitable for use in murine experiments using *T congolense* or *T*. *brucei* species of trypanosomes with IP as most suitable for pathogenicity studies. However, the SC is superior to the ID, IM and IV routes in that it is easy to administer and yet less painful [41]. It is also applicable to a conscious animal making it therefore a comparable alternative of the IP route on maurine trypanosomes experiments. In this study, we used only one *T*. *brucei* and *T*. *congolense* isolates and there will be need to confirm our observation by increasing the number of the isolates.

## Supporting information

S1 Checklist (DOCX)

S1 Data summary (XLSX)
