# Supplementary material for: Route of inoculation influences Trypanosoma congolense and Trypanosoma brucei brucei virulence in Swiss white mice
Source: PLoS One. 2019 Jun 20;14(6):e0218441. doi: 10.1371/journal.pone.0218441 (PMC6586304; doi:10.1371/journal.pone.0218441)
Supplement: S1 Checklist — (DOCX) [file pone.0218441.s001.docx]

**ARRIVE Guidelines checklist**

| **ITEM** |  | **Response** |
| --- | --- | --- |
| Title | 1 | Route of inoculation influences *Trypanosoma congolense* and *Trypanosoma brucei brucei* virulence in Swiss white mice |
| Abstract | 2 | **Background:** Experiments on infections caused by trypanosomes are widely performed in Swiss white mice through various inoculation routes. To better understand the effect of route of trypanosome inoculation on disease outcomes in this model, we characterised the virulence of two isolates, *Trypanosoma bruce*i KETRI 2710 and *T.* *congolense* KETRI 2765.  **Experimental approach:** Swiss white mice were injected with each of the isolates through five routes of parasite inoculation, namely intraperitoneal (IP), subcutaneous (SC), intramuscular (IM) intradermal (ID) and intravenous (IV) and compared using groups (n = 6) of mice. Each mouse received 1x10^4^ trypanosomes. We assessed impact of the routes on disease indices that included pre-patent period (PP), parasitaemia levels, Packed Cell Volume (PCV), bodyweight changes and survival time  **Key results:** Pre-patent period for IP inoculated mice was a mean ± SE of 3.8 ± 0.2 and 6.5 ± 0.0 for the *T brucei* and *T. congolense* isolates respectively; the PP for mice groups inoculated using other routes were not significantly different(p> 0.05) irrespective of route of inoculation and species of trypanosomes. With ID and IP routes, parasitaemia was significantly higher in *T. brucei* and significantly lower in *T. congolense* infected mice and the progression to peak parasitaemia routes showed no significant different between the routes of either species of trypanosome .The IM and ID routes in *T. congolense* inoculations, and IP and IV in *T. b. brucei* induced the fastest and slowest parasitaemia progressions respectively. There were significant differences in rates of reduction of PCV with time post infection in mice infected by the two species and which was more pronounced in sc and ip injected mice. No significant differences in mice body weight changes and survivorship was observed between the routes of inoculation.  **Conclusions and implications:** Inoculation route appears to be a critical determinant of pathogenicity of *Trypanosoma congolense* and *Trypanosoma brucei brucei* in murine mouse model of African trypanosomiasis. |
| **Introduction** | | |
| a)Background | 3 | African trypanosomes are extracellular protozoan parasites that cause chronic infections in Humans and their livestock, and are predominantly transmitted by tsetse fly[[1](#_ENREF_1)][.](#page18) *Trypanosoma congolense and Trypanosoma brucei brucei*, are among the main trypanosomes, which causes livestock infections [[2](#_ENREF_2),[3](#_ENREF_3)].Available data on *T. congolense* and *T. b. brucei* pathogenicity suggest *T. congolense* as the most pathogenic trypanosome in cattle [[4](#_ENREF_4)] and has been shown to exhibit greater virulence in experimental mice [[5](#_ENREF_5)][.](#page19) In contrast, *T. b.* *brucei* is particularly virulent in dogs, camels and horses, with the horse often succumbing to infection within a few months in the absence of treatment[[6](#_ENREF_6)] The pathogenicity of these parasites has typically been determined through intraperitoneal (IP) inoculation[[7](#_ENREF_7),[8](#_ENREF_8)] of Swiss white mice animal models of trypanosomiasis[[9](#_ENREF_9)] Parasitaemia, live body weight, packed cell volume (PCV) and survivorship in the mice have been considered appropriate indicators of the pathogenicity[[5](#_ENREF_5)] [[10](#_ENREF_10)].The various routes of inoculation including intraperitoneal (IP),subcutaneous(SC), intramuscular (IM), intravenous (IV) and intradermal (ID) have, previously been used to infect mice with trypanosomes[[11](#_ENREF_11),[12](#_ENREF_12),[13](#_ENREF_13)] The IP inoculation is normally administered through the abdominal wall into the peritoneal cavity of the mice. Consequently, there is no visual confirmation of inoculation as opposed to other comparable methods such as intravenous (IV) or intradermal (ID) routes [[14](#_ENREF_14)] While IV has been used as an inoculation route, the technique exposes mice to higher risks of infection by other pathogens [[15](#_ENREF_15)]and tissue irritation in mice. Similarly, intramuscular (IM) route has an advantage of rapid and uniform deposition of the inoculum[[15](#_ENREF_15)] but institutes muscle necrosis or inflammation of the nerves that can lead to lameness and self-mutilation of the affected area[[16](#_ENREF_16),[17](#_ENREF_17)].Administration of ID inoculation is complicated by thin skin in mice that necessitates anesthetization and the fur clipping of the mice[[18](#_ENREF_18)] with inadvertent SC administration as a common complication [[7](#_ENREF_7)][.](#page19) |
| b) Animal model |  | For this purpose we used the Swiss White Mice animal model for it at least show outcomes similar to African trypanosome infection in other mammals. The model will allow us to robustly address the set research questions, corroborate our outcomes with other studies and potentially allow future investigations. Our desired outcomes can be best realised using a mouse model |
| Objective | 4 | The objective of this study, was to compare the impact of route of inoculation of *T. congolense* KETRI 2765 or *T. b. brucei* KETRI 2710 parasites in Swiss white mice on pathogenicity of the parasites. |
| **Methods** | | |
| Ethical statement | 5 | All protocols and procedures used in this study were reviewed and approved by the  Biotechnology Research Institute Kenya Agricultural and Livestock Research Organization  (BioRI-KALRO-) Institutional Animal Care and Use Committee (IACUC) licence number C/BioRI/4/325/II/47 |
| Study design | 6 | **a)Groups :**Five groups of six mice each were each were intraperitoneally, subcutaneously, intramuscularly, intradermally or intravenously inoculated with 0.1mL (1.0 x 10^4^ trypanosomes) of the *T. brucei* or *T. congolense* infected blood in PSG. Six mice were similarly handled but were not inoculated with any of the parasites and therefore constituted the control group for our inoculated groups.  **b) Animal selection**: Mice were selected and housed in plastic cages based on their body weights which ranged between 25-30g per mouse.  **c) Number of animals:** In this experiment, n refers to number of animals in a cage referred to as a group with each mouse in this group injected with trypanosomes through one of the routes under study. |
| Experimental procedures | 7 | \| **Activity** \| **Wk1** \| **Wk 2** \| **Wk 3** \| **Wk 4** \| **Wk 5** \| **Wk 6** \| **Wk 7** \| \| --- \| --- \| --- \| --- \| --- \| --- \| --- \| --- \| \| 1. Acquire animal \|  \|  \|  \|  \|  \|  \|  \| \| 2. Donor mice Immunosuppression \|  \|  \|  \|  \|  \|  \|  \| \| 3. Inject donor mice \|  \|  \|  \|  \|  \|  \|  \| \| 4 .Experimental Animals acclimatization \|  \|  \|  \|  \|  \|  \|  \| \| 5. Donor mice parasitaemia monitoring \|  \|  \|  \|  \|  \|  \|  \| \| 6. Infect experimental mice \|  \|  \|  \|  \|  \|  \|  \| \| 7. Monitor infected experimental animals \|  \|  \|  \|  \|  \|  \|  \| |
|  |  | **Preparation of inoculum dose**: At peak parasitaemia (1.3x10^8^ trypanosomes/ml for the *T. congolense* and 5.0x10^8^trypanosomes/ml for the *T. b. brucei)*, the donor mice were anaesthetized using carbon dioxide, the parasites collected through cardiac puncture in tubes containing 10% EDTA and quantified using improved Neubauer chamber method of [[23](#_ENREF_23)] The parasites (in blood) were subsequently diluted to 1.0 x 10^4^ trypanosomes/mL with Phosphate-buffered-Saline-Glucose (PSG) (44 mM NaCl, 57 mM Na_2_HPO_4_, 3 mM KH_2_PO_4_, 55 mM glucose) pH 8.0 solution. Subsequently, five groups of six mice [[5](#_ENREF_5),[24](#_ENREF_24),[25](#_ENREF_25)]each were intraperitoneally, subcutaneously, intramuscularly, intradermaly or intravenously inoculated with 0.1mL (1.0 x 10^4^ trypanosomes) of the *T.brucei* or *T. congolense* infected blood in PSG |
|  |  |  |
| Experimental animals | 8 | **Animal:** Swiss white male mice (25-30g) were used in this study (n=70)  **Source**: Seventy mice were obtained from BioRI-KALRO Small Animals Breeding Unit |
| Housing and husbandry | 9 | **Housing:** Animals were housed in plastic cages (6 per cage) measuring 30x13 cm and were kept under natural light without controlled temperature and humidity.  **Husbandry:** All mice were allowed free access to water and maintained on mice pellets (Unga Feeds Ltd, Kenya) constituted of crude protein (18.1%), Calcium minimum (0.8%), Phosphorus min (0.8%), Fat maximum (8%) and crude fibre (7%).  **Animal welfare:** To avoid exsanguination, infected mice were monitored daily for parasitaemia development using the tail artery/vein bleed (tail nick bleed) method where the mouse was firmly restrained, the tail sterilised using 70% alcohol and a using a sample pair of scissors, the tip end of the tail cut and about 20 µl blood collected onto a clean slide and covered with a coverslip. The endpoint of the infected mice were determined by observation of clinical signs such as lethargy and hackle hair, as well as PCV drop of approximately 25% with consistent high parasitaemia levels of 1 × 10^9^/ml for at least 3 consecutive days. The animals were sacrificed immediately by CO_2_ asphyxiation in accordance with guidelines of the Institutional Animal Care and Use Committee (IACUC) and as described by (Martinez-Gutierrez et al. 2014) and recorded as dead animals. |
| Sample size | 10 | **Number of animals used**: Seventy healthy mice were divided into four groups: group1; 4 donor mice for the propagation of *T. congolense* and *T. Brucei*. Group 2; thirty mice in five groups of 6 mice each infected with *T. congolense* through the five routes of injection. Group 3; thirty mice similarly grouped and infected with *T. brucei* as was with *T. congolense*. Group 4; Six non-infected mice serving as the control for the *T. congolense* and *T. brucei* infected mice.  **Arrival at number of animals:** The choice of the number of mice per group was influenced by data generated from previous studies using the same sample size ([Janelle J](https://www.ncbi.nlm.nih.gov/pubmed/?term=Janelle%20J%5BAuthor%5D&cauthor=true&cauthor_uid=19720159), et al, 2009, Kariuki C et al, 2015 and Gitonga et al, 2017). |
| Allocating animals to experimental groups | 11 |  |
| Experimental outcomes | 12 | Six primary outcome measures were analysed. These were pre-patent period (PP), parasitaemia progression, peak parasitaemia, packed cells volume (PCV), body weight changes and survivorship. |
| Statistical methods | 13 | Differences in pre-patent periods and effect of inoculation routes and trypanosome species on peak parasitaemia were evaluated using two way analysis of variance (ANOVA) with inoculation routes and parasite species as factors. Means that were significantly different were identified using Tukey’s HSD post hoc analysis[[30](#_ENREF_30)]. Differences in rates of increase in *T. congolense* or *T.b brucei* parasitaemia following inoculations through various routes were established by comparing effective median times (ET50) for each inoculation route using Probit analysis. Effects of different inoculation routes on PCV and bodyweights were analysed through linear regression analysis of the changes in weight of individual mice against time (days). Impact of the different of inoculation routes on survivorship of *T. congolense* or *T. b brucei* were analyzed by Kaplan-Meier method to determine survival distribution [[31](#_ENREF_31)]function with log-rank (Mantel-Cox) and Gehan-Breslow-Wilcoxon test[[32](#_ENREF_32)] to provide insight on effect of route of trypanosome infection on survivorship. All analyses were conducted using GraphPad Prism version 7.00 for Mac (GraphPad Software, La Jolla California USA). |
| **Results** | | |
| Baseline data | 14 | Prior to infection, experimental mice were acclimatized to experimental room conditions for two weeks during which period they were dewormed by intraperitonial injection using Ivermectin (Noromectin®) at 0.2mg/kg as described by[[19](#_ENREF_19)] to potentially eliminate possible ecto- and endoparasites in the mice. Base line data on PCV and body weight changes were collected and recorded. The animals’ health status was monitored by a veterinarian working for the institution |
| Numbers analysed | 15 | *T. congolense* infection: 6/6 (n=6) of mice infected with via IM, SC and ID; 3/6 and 4/6 of mice infected via IV and IP respectively.  *T. brucei* infection: (n=6) mice inoculated via IP, IM, SC and IV and (5/6) of the mice inoculated via ID route.  **Data not included in the analysis:** 3/6, 2/6 of *T. congolense* infected mice via IV and IP and 1/6 of *T. brucei* infected mice via ID routes. These mice did not develop parasitaemia throughout the monitoring period. |
| Outcome and estimation | 16 | The pre-patent periods within mice infected with either parasite were similar, irrespective of the routes on inoculation (F4, 44 = 2.03, P=0.1067). The overall peak parasitaemia in either species was not significantly influenced by the route of inoculation (F4, 48 = 0.7201, P = 0.5824). The mode of inoculation significantly influenced the progression (ET50) of *T. congolense* (F4, 637= 32.11, P = <0.0001) or *T. b. brucei (*F4, 382 = 8.903, P =<0.0001) infections in mice. The IM and ID routes in *T. congolense* inoculations, and IP and IV in *T. b. brucei* induced the fastest and slowest parasitaemia progressions respectively (Fig B (i) &(ii)). There were significant differences in rates of reduction of PCV (F5, 14= 4.632, P =0.0105) with time post infection in mice infected by the two species and which was more pronounced in sc and ip injected mice (Table 1) (F 1, 3 = 10.36, P = 0.0486). However, no significant differences in bodyweight was observed among different routes of injection (Table 2). The routes of inoculation significantly influenced survivorship in mice inoculated by *T. congolense* (Χ^2^ (df = 5) = 11.35, p = 00449) or *T. b. brucei* (Χ^2^ (df = 5) = 18.83, p = 0021), with *T. congolense* inoculated mice living longer (Fig C (i)&(ii)). However, there was no significant difference between the routes of either group. |
| Adverse events | 17 | No adverse effect |
| **Discussion** | | |
| Interpretation/ scientific implications | 18 | In this study, we investigated the effect of intraperitonial (IP), subcutaneous (SC), intramuscular (IM), intravenous (IV) and intradermal (ID) routes of inoculation on the pathogenesis of *T. congolense* or *T. brucei* trypanosomes infection in the murine model. In our current study, we obtained a complete 6/6 infection success rate for all groups with the exception of IP- *T congolense* group which had 4/6 (67%), IV -*T congolense* group with 3/6 (50%), and ID-*T. brucei* group with 5/6 (83%) demonstrating the effectiveness of these routes in experimental trypanosomiasis. Data from our study showed occurrence of anaemia in mice infected with *T. congolense* or *T. brucei* irrespective of the route of inoculation and in agreement with previous observation[[38](#_ENREF_38)][.](#page20) However, the decline in PCV was not significant suggesting all the routes are ideal for inoculation in studies on anaemia induced by either of these species of trypanosomes. Data from our study showed that the route of inoculation influenced the survival of the infected mice. However, there was no significant difference between the routes of inoculation in mice infected by the two species of trypanosomes suggesting therefore none of the routes is superior in virulence studies[[5](#_ENREF_5)] [[40](#_ENREF_40)] using the two species of trypanosomes.  **Study limitation**: In this study, we used only one *T. brucei* and *T. congolense* isolates and there will be need to confirm our observation by increasing the number of the isolates |
| Generalisability/ translation | 19 | In conclusion, our study showed that all the routes are suitable for use in murine experiments using *T congolense* or *T. brucei* species of trypanosomes with IP as most suitable for pathogenicity studies. However, the SC is superior to the ID, IM and IV routes in that it is easy to administer and yet less painful [[41](#_ENREF_41)]. It is also applicable to a conscious animal making it therefore a comparable alternative of the IP route on maurine trypanosomes experiments |
| Funding | 20 | The research leading to these results was funded by the Kenya Government through the Kenya Agricultural and Livestock Research Organization (KALRO)-Biotechnology Research Institute (BioRI). |
